# Contribution to the characterization of the seed endophyte microbiome of *Argania spinosa* across geographical locations in Central Morocco using metagenomic approaches

**DOI:** 10.3389/fmicb.2024.1310395

**Published:** 2024-03-25

**Authors:** Hourfane Sohaib, Morgan Fays, Abderrezzak Khatib, John Rivière, Noureddine El Aouad, Nicolas Desoignies

**Affiliations:** ^1^Laboratory of Life and Health Sciences, Faculty of Medicine and Pharmacy of Tangier, University Abdelmalek Essaâdi, Tetouan, Morocco; ^2^Phytopathology, Microbial and Molecular Farming Lab, Centre D’Etudes et Recherche Appliquée-Haute Ecole Provinciale du Hainaut Condorcet, Ath, Belgium; ^3^Laboratory of Biotechnology and Applied Biology, Haute Ecole Provinciale de Hainaut-Condorcet, Ath, Hainaut, Belgium; ^4^University of Liege - Gembloux Agro-Bio Tech, TERRA - Teaching and Research Center, Plant Sciences Axis, Gembloux, Belgium

**Keywords:** *Argania spinosa*, bacteria, diversity, endophytes, fungus, NGS, PCR, seed

## Abstract

Microbial endophytes are microorganisms that live inside plants, and some of them play important yet understudied roles in plant health, growth, and adaptation to environmental conditions. Their diversity within plants has traditionally been underestimated due to the limitations of culture-dependent techniques. Metagenomic profiling provides a culture-independent approach to characterize entire microbial communities. The argan tree (*Argania spinosa*) is ecologically and economically important in Morocco, yet its seed endophyte microbiome remains unexplored. This study aimed to compare the bacterial and fungal endophyte communities associated with argan seeds collected from six sites across Morocco using Illumina MiSeq sequencing of the 16S rRNA gene and ITS regions, respectively. Bacterial DNA was extracted from surface-sterilized seeds and amplified using universal primers, while fungal DNA was isolated directly from seeds. Bioinformatics analysis of sequencing data identified taxonomic profiles at the phylum to genus levels. The results indicated that bacterial communities were dominated by the genus *Rhodoligotrophos*, while fungal communities exhibited varying degrees of dominance between *Ascomycota* and *Basidiomycota* depending on site, with *Penicillium* being the most abundant overall. Distinct site-specific profiles were observed, with *Pseudomonas*, *Bacillus,* and *Aspergillus* present across multiple locations. Alpha diversity indices revealed variation in endophyte richness between seed sources. In conclusion, this first exploration of the argan seed endophyte microbiome demonstrated environmental influence on community structure. While facing limitations due to small sample sizes and lack of ecological metadata, it provides a foundation for future mechanistic investigations into how specific endophyte–host interactions shape argan adaptation across Morocco’s diverse landscapes.

## 1 Introduction

Endophytes are microorganisms that reside asymptomatically within plant tissues and form intricate relationships with their host plants (Petrini, 1991). They play multiple roles in plant growth promotion, stress tolerance, secondary metabolite production, and nutrient recycling (Higa and Parr, 1994; Santos and Olivares, 2021). Endophytes can be found on many plant parts ranging from the roots to the seeds, and the roles of seed endophytes have been well studied. For instance, *Kosakonia cowanii* identified on the seeds of *Lactuca serriola* was found to produce extracellular polysaccharides, aiding in maintaining soil moisture (Jeong et al., 2021). Other endophytes improve the response of plants to external biotic or abiotic stressors, such as saline stress of *Medicago sativa*, and soybean, which are enhanced by *Pontoea* sp., *Bacillus* sp., and *Fusarium verticillioides* (Radhakrishnan et al., 2013; Truyens et al., 2014). Insect herbivory against *Festuca rubra* is reduced by alkaloids produced by *Epicholë festucae* (Laihonen et al., 2022), and resistance to pathogens is induced by *Paenibacillus dendritiformis* in *Zea maize* plants (Pal et al., 2022).

Many studies have reported the qualitative and quantitative endophytic composition of Moroccan plants (Debbab et al., 2009; Imane et al., 2018). Some of these articles focus on endemic species such as *Argania spinosa* (Abdallah et al., 2019; Mapelli et al., 2020). The argan tree (*Argania spinosa*) is an arboreous plant belonging to the Sapotaceae family. It is an emblematic plant endemic to Morocco that has adapted to harsh desert conditions through interactions with its microbiome (Mechqoq et al., 2021a). As a foundational source of argan oil and keystone species supporting biodiversity (Mechqoq et al., 2021b), further elucidating the argan microbiome could uncover applications in sustainable agriculture, medicine, and ecology.

In fact, understanding the significance and potential roles of *Argania spinosa* seed microbial population is crucial for designing sustainable management strategies, conservation efforts, and agricultural practices related to argan cultivation (Mapelli et al., 2020). By harnessing the beneficial properties of seedborne microbes, it may be possible to improve seed quality, enhance plant growth and health, and promote the long-term survival of *Argania spinosa* populations in their natural habitats or in agricultural settings (Ganoudi et al., 2021). In addition to its ecological significance, the seedborne microbial population of *Argania spinosa* may have potential implications in medicine and human health. While research in this specific area is limited, there is growing interest in exploring the medicinal properties of plants and their associated microbial communities.

The seedborne microbial population can produce bioactive compounds with therapeutic properties, including antimicrobial agents, antioxidants, anti-inflammatory, anticancer, or immunomodulatory substances, which could be utilized for new drug development or natural remedies (Hashem et al., 2023), as well as dermatological applications. Those microorganisms may influence the production of bioactive compounds or enzymes in argan oil (Balogun et al., 2023), known for its beneficial effects on the skin and hair (Mechqoq et al., 2021b), potentially leading to the development of new skincare or haircare products by understanding the microbial composition of the seed.

Traditionally, endophytes are investigated using culture-based studies that provide partial snapshots biased toward fast-growing isolates (Moore et al., 2006). Recent advances such as metagenomics now enable direct profiling of entire microbial populations within plants (Hassa et al., 2018). Mechanistic research has also begun deciphering the specific modes by which endophytes enhance growth via secreting compounds, nutrient recycling, and abiotic stress mitigation (Wani et al., 2022). However, the functional potentials and interactive networks of argan endophytes remain obscure.

To preliminarily address these gaps and form a foundation for future studies, this study employed a culture-independent approach analyzing bacterial and fungal DNA extracted from argan seeds collected across six sites in Morocco. Rather than a comprehensive survey, the aim was to generate novel population data and examine community variation between seed sources. While the small sample size and isolation of a single timepoint limited robust conclusions, the results provide empirical leads worth deeper exploration.

## 2 Materials and methods

### 2.1 Description of the research area

This study had been limited to the Moroccan west-central area, in the vicinity of Agadir along the Atlantic coast. This area is characterized by its semi-arid climate with hot summers and mild winters. In April, average temperatures vary between 20°C and 25°C, with average annual rainfall ranging from 200 to 400 mm. This area is the homeland of the argane grove known as “*arganeraie*,” and this grove stretches from the mouth of the Tensift river in the north, the mouth of the Drâa river in the south, the southern slopes of the western High Atlas to the east, and the northern and southern slopes of the western Anti-Atlas (Msanda et al., 2005).

### 2.2 Sample collection and preparation

The argan fruits were harvested from six different sites marked as T1, T2, T3, T4, T5, and T6. For each locality, healthy trees were targeted, and fruits with relatively similar color, caliber, and healthy aspects were chosen. The collection sites (T1, T4, T5, and T6) were located at least 50 km apart. However, sites T2 and T3 were in closer proximity at approximately 3 km separation due to their locations on different landscape features (T2 in a plain and T3 near a mountain). Their inclusion was deliberate, aiming to test for potential edaphic and topographic influences on endophyte community composition over a small spatial scale.

**Figure 1:**
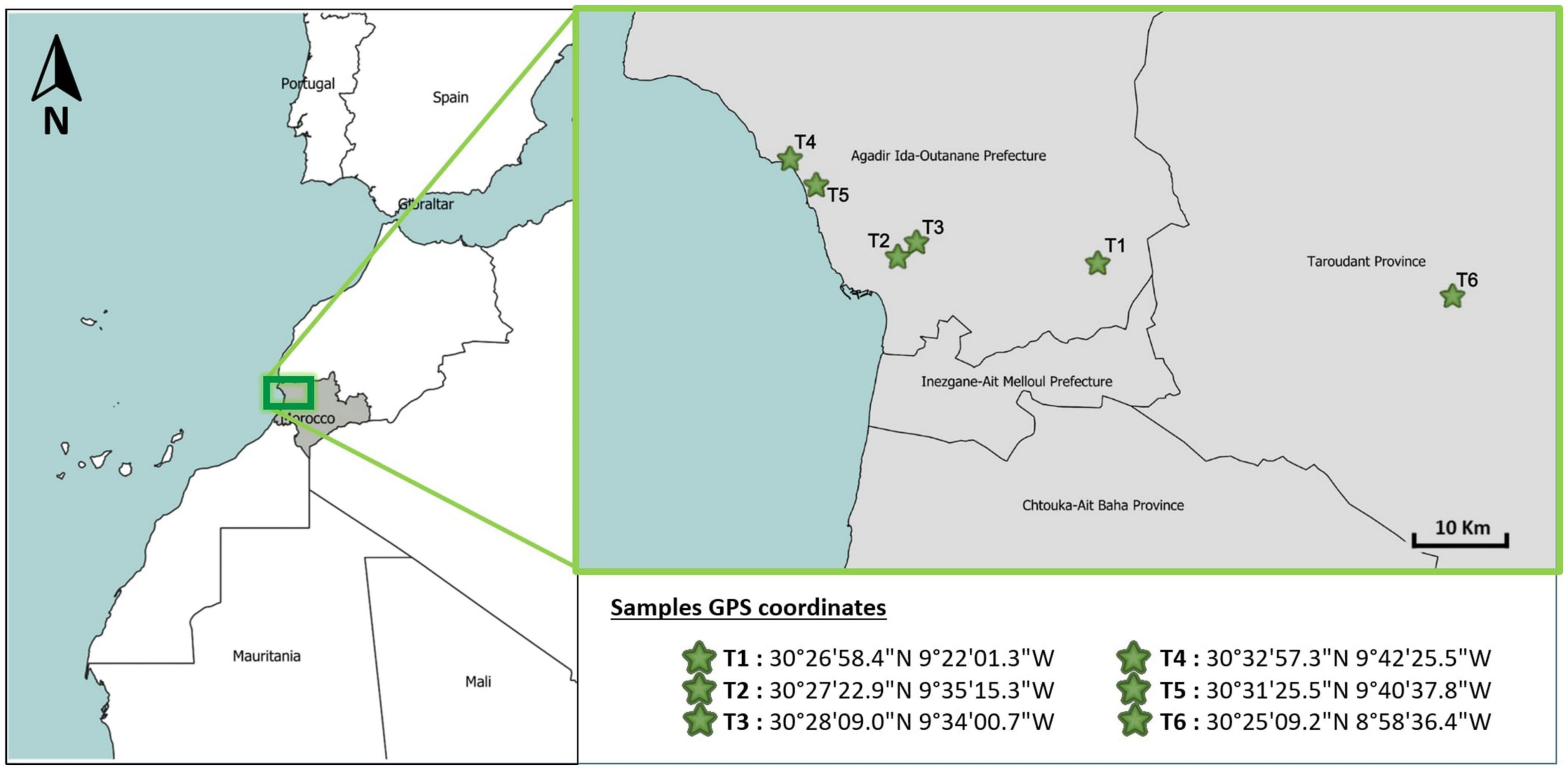
Geographical distribution of sampling points.

The global positioning system (GPS) coordinates corresponding to the respective collection sites of the samples are displayed in Figure 1. Seed sampling was carried out along a single road that wound through the six geographically separated sampling sites. At each site, the road was followed, and 10 argan trees located a minimum of 50 m apart were selected randomly for sampling. To collect seeds from each tree, a pruning pole equipped with a clipper was used to gather fallen seeds from the ground surface beneath the canopy without disturbing the tree. At each site, 10 trees were randomly selected, totaling 60 trees across all sites. From each tree, 3 healthy seeds were collected aseptically, resulting in a total of 180 seeds (30 seeds × 6 sites) by slowly traveling the road and randomly selecting seeds from trees along the way.

Seeds collected in the field were subjected to a surface sterilization protocol under sterile conditions within a biosafety cabinet. The aim of this protocol was to eliminate external microbes from the seed surface in order to avoid cross-contamination during subsequent DNA extraction and analysis of internal endophytic communities. Individual seeds were placed in a sterile glass jar filled with 100 mL of 1% Triton X-100 solution for 5 min with gentle agitation. The seeds were then transferred using sterile forceps to a glass jar containing 100 mL of a 70% ethanol solution and shaken at 100 rpm for 2 min to remove contaminants. After ethanol treatment, the seeds were transferred to a glass jar containing 30 mL of a 2° sodium hypochlorite (bleach) solution and shaken at 100 rpm for 10 min to disinfect and kill any remaining microbes. Next, the seeds were rinsed by transferring them to three successive Petri dishes of sterile distilled water, with agitation to remove residues from the previous solutions. Finally, the surface-sterilized seeds were used immediately for DNA extraction and metagenomic sequencing or stored at −20°C.

### 2.3 Metagenomics with next-generation sequencing

#### 2.3.1 Isolation of total genomic DNA

DNA was extracted from plant seeds to analyze their endophytic microbiome. The endophytic microbiome of each seed was extracted and sequenced individually. DNA extractions were performed using a FastDNA SPIN soil kit (Qbiogene Inc., Carlsbad, CA, United States) according to the manufacturer’s instructions. All DNA extractions were performed using the same FastDNA SPIN dish to minimize the potential effect of extraction kit contamination.

DNA quality was assessed by 1% agarose gel electrophoresis, and DNA concentrations were determined using a NanoDrop 3,300 Fluorospectrometer (Thermo Fisher Scientific Inc., Waltham, MA) with PicoGreen reagent (Invitrogen, Carlsbad, CA). Prior to the PCR, the DNA concentration of all samples was normalized to 50 ng/μL. Normalizing DNA concentrations prior to PCR helps ensure comparable amplification efficiency across samples and reduces potential bias.

#### 2.3.2 Amplification of the bacterial and fungal target

To confirm the success of the DNA extraction from the seed pellet, DNA quantification and polymerase chain reaction (PCR) assays of the target gene were performed. Bacterial community structure was determined by sequencing the V3–V4 variable region of the 16S rRNA gene. Libraries were prepared according to the 16S metagenomic sequencing library preparation protocol (Illumina) using the primer pair 16SV3V4F (5′- TCGTCGGCAGCGTCAGATGTGTATAAGAGACAGCCTACGGGNGGCWGCAG- 3′) and 16SV3V4R (5’-GTCTCGTGGGCTCGGAGATGTGTATAAGAGACAGGACTACHVGGGTATCTAATCC- 3′) with an average fragment length of 460 bp under the following conditions: 98°C for 30 s, followed by 25 cycles of 98°C (10 s), 55°C (30 s), and 72°C (30 s), with a final extension at 72°C (10 min) (Amplicon et al., 2013; Yu et al., 2022). The fungal community was also determined by sequencing ITS2 using a wide range of primers: ITS3_KYO1_Illumina (5’-TCGTCGGCAGCGTCAGATGTGTATAAGAGACAGAHCGATGAAGAACRYAG-3′) (Toju et al., 2012) and ITS4_Illumina (5’-GTCTCGTGGGCTCGGAGATGTGTATAAGAGACAGTCCTCCGCTTATTGATAT GC- 3′) (Toju et al., 2012) with an average fragment length of 300–350 bp under the following conditions: 98°C for 30 s, followed by 25 cycles of 98°C (10 s), 53°C (30 s), and 72°C (30 s), with a final extension at 72°C (10 min). PCR reactions were performed in a final volume of 20 μL containing 10 μL of Phusion Plus Master Mix (Promega, Madison, WI, United States), 1 μL of each primer (10 μM), 3 μL of DNA template (~1 ng per μl), and 5 μL of nuclease-free water. PCR products were then verified by electrophoresis on a 1% agarose gel.

#### 2.3.3 Indexing and amplification

Following the PCR amplification of the internal transcribed spacer region 2 (ITS2) and V3–V4 hypervariable region of the 16S rRNA gene, the resulting amplicons were separately purified and indexed using the Nextera XT Index kit (Illumina). Indexing was performed via a second PCR involving a 50-μl reaction mixture containing 25 μL of Phusion Plus Master Mix, 5 μL of index primers, 5 μL of DNA template from the first PCR, and 10 μL of nuclease-free water. The thermocycling conditions were as follows: initial denaturation at 95°C for 3 min, followed by 8 cycles of 95°C for 30 s, 55°C for 30 s, and 72°C for 30 s, and a final extension at 72°C for 10 min.

#### 2.3.4 Purification and quantification

After indexing, the samples (DNA libraries) underwent a final purification step using SparQ PureMag beads (Quantabio, VWR) according to the manufacturer’s instructions. Moreover, the quality and quantity of the libraries were assessed using the PicoGreen kit (Thermo Fisher Scientific) and the TapeStation band analyzer (Agilent).

#### 2.3.5 Pooling libraries—concentration and standardization

Prior to pooling the samples into libraries, the concentration of each amplicon was measured using a NanoDropTM 2000/c spectrophotometer (Thermo Fisher Scientific, Vienna, Austria). Then, amplicons were diluted with resuspension buffer to achieve a normalized concentration of 4 nM. Samples were pooled in equimolar amounts to create the amplicon libraries. The concentration of the libraries in nM was determined following the protocol of the NEBNext Ultra DNA Library Prep Kit for Illumina (New England Biolabs, Ipswich, MA, United States). A final library concentration of 4 pM was utilized for sequencing.

#### 2.3.6 Sequencing and bioinformatics analysis

The prepared libraries were sequenced on an Illumina MiSeq platform using the 2×300 sequence protocol in paired-end mode (MiSeq Reagent Kit v3-600 cycles; Illumina, San Diego, CA, United States). Fastq files were generated for each sample (demultiplexed) using MiSeq software (Illumina Inc., San Diego, CA, United States). In addition, raw metagenomic datasets were deposited in the NCBI Sequence Read Archive database under BioSample accessions SAMN40034098, SAMN40034099, SAMN40034100, SAMN40034101, SAMN40034102, SAMN40034103, SAMN40034104, SAMN40034105, SAMN40034106, SAMN40034107, SAMN40034108, and SAMN40034109. Furthermore, bioinformatics analysis was performed using an in-house pipeline to obtain a taxonomic assignment of the sequences obtained and a count of the sequences obtained for each sample. Our in-house pipeline is based on various bioinformatics tools (Cutadapt (Martin, 2011), Pear (Zhang et al., 2014), DADA2 (Callahan et al., 2016), Uchime (Edgar et al., 2011)) and includes the following major steps to generate a csv file: Primer cutting, pre-assembly—Use of the DADA2 package—Elimination of chimeric sequences—Taxonomic attribution with respect to the BLAST databases (NCBI). Taxonomic data are normalized in relative abundance for each sample and then analyzed with graphs.

### 2.4 Statistical analysis

Measures of alpha diversity including the Shannon, Chao, and Simpson indices and other parameters, such as principal component analysis (PCA) and relative abundance (%), were applied to evaluate the diversity of the argan seed microbiomes. These analyses were performed using PAST 4.03 software (Hammer, University of Oslo, Oslo, Norway) (Hammer et al., 2001). Graphs were created using Microsoft Excel version 16.0 (Microsoft Corp., Redmond, WA, United States) (Carr, 2008) and R Studio version 6.1 (RStudio Inc., Boston, MA, United States) (Gandrud, 2018), with the R packages ggplot2 (Valero-Mora, 2010) and gridExtra (Auguie et al., 2017) used to create figures.

The maps were generated using QGIS version 3.32.1 (QGIS Development Team, Brno, Czech Republic) (Kurt Menke et al., 2016). Furthermore, the Venn diagram of the operational taxonomic units (OTUs) identified on the samples was drawn using the online servers VennWebTool (accessed 04 August 2023)^1^ and JVenn (accessed 04 August 2023)^2^.

## 3 Results

### 3.1 Microbial structure, diversity, and richness

#### 3.1.1 Bacterial community

**Figure 2:**
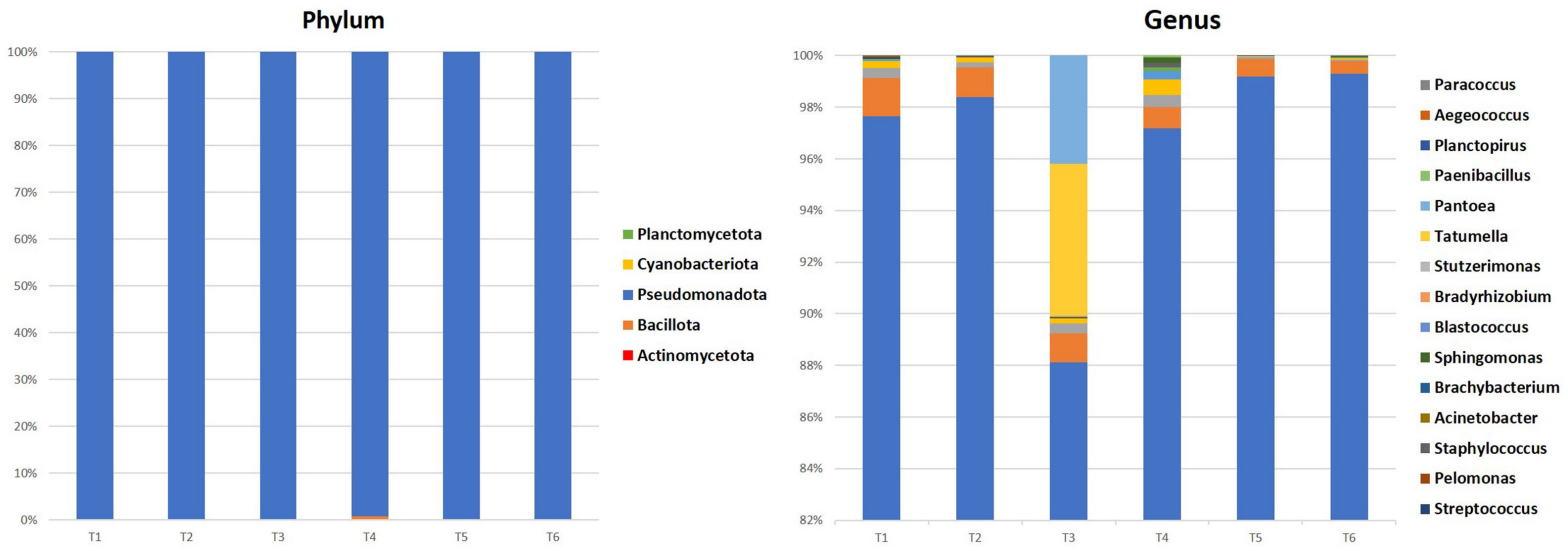
Percentages of phylum and the 10 most representative bacterial genera in all samples.

The metagenomic analysis of 16S rRNA gene amplicons reveals endophytic bacterial communities’ diversity within argane seeds. Figure 2 provides a visual summary of the relative abundances of major bacterial phyla and genera within each seed sample, highlighting the community variations and niche differentiations between territories at different taxonomic levels.

At the phylum level, *Pseudomonadota* dominated all seed samples accounting for 99.4–99.85%, followed by Bacillota and *Actinomycetota* with respective values ranging between 0.01–0.6 and 0.01–0.15%. At the genera level, *Rhodoligotrophos* was the most prevalent and abundant across most seed samples, comprising between 88 and 99% of bacteria. The highest value was observed in territory 6 (T6) with 99.29%, while the lowest value was observed in territory 3 (T3) with 88.04%. Other genera such as *Pseudomonas*, *Paraburkholderia*, *Ralstonia*, *Bacillus,* and others were also detected, though in varying proportions depending on the seed’s territory of origin.

**Figure 3:**
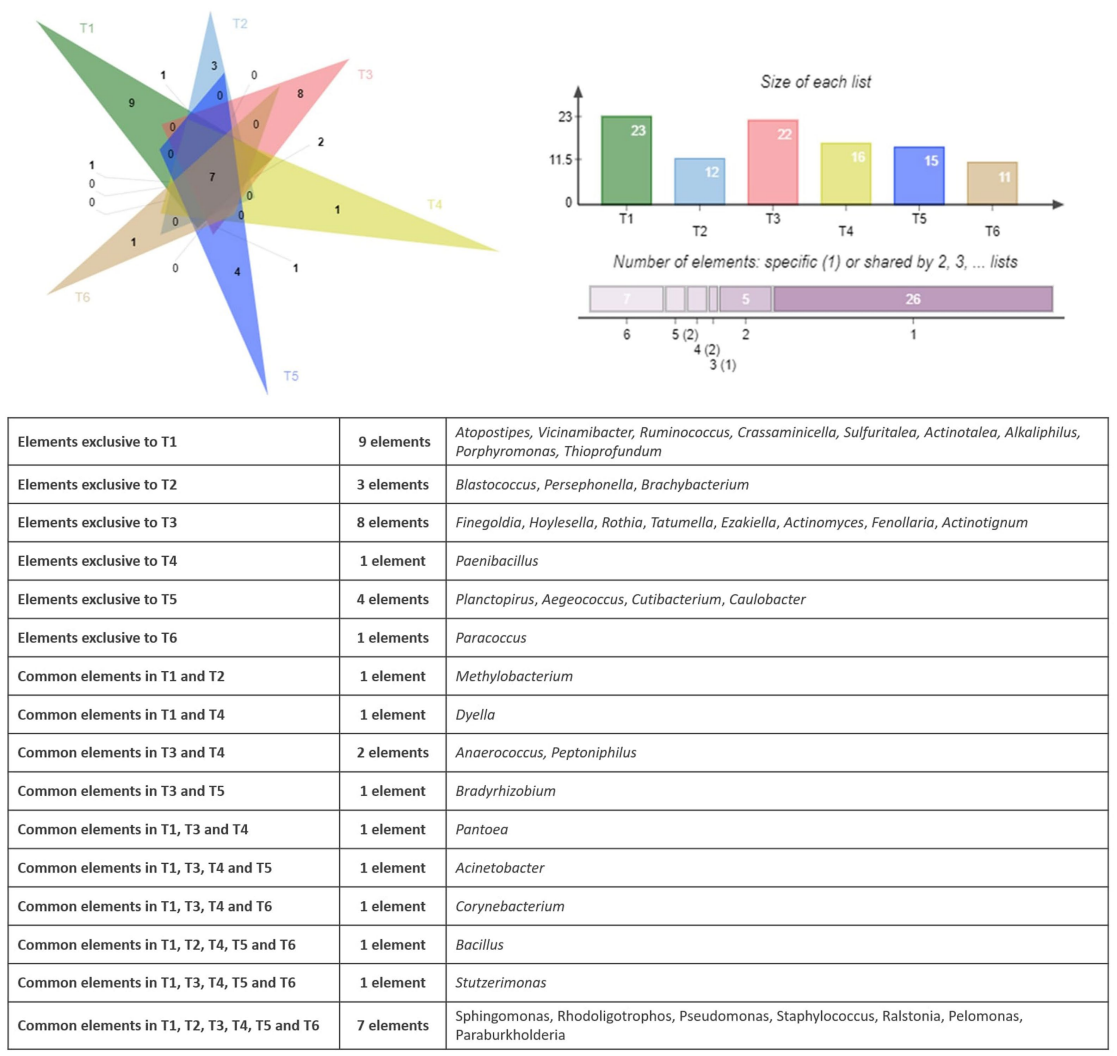
Venn diagram showing the specific and common genera of the bacterial community.

Figure 3 shows the shared and unique bacterial genera identified in the endophytic microbiome of argan seed samples collected from six different territories. Each triangle represents the set of genera found in seeds from one territory. The lighter colored regions indicate genera that are uniquely present in the seed samples from that territory while not found in the other territories. The darker colored regions (crossing triangles) show genera that are shared between two or more territories.

The observation of the bacterial genera distribution in the six territories (T1 to T6) shows that there are both ubiquitous and specific genera in certain territories. Seven bacterial genera had been identified in all the territories namely *Sphingomonas, Rhodoligotrophos, Pseudomonas, Staphylococcus, Ralstonia, Pelomonas, and Paraburkholderia*. When some genera are exclusive to a few territories, such as, *Anaerococcus and Peptoniphilus* in T3 and T4, *Bradyrhizobium* in T3 and T5, *Dyella* in T1 and T4, and *Pantoea* in T1, T3, and T4, other genera are exclusive to one territory, such as *Paenibacillus* found in T4 and *Paracoccus* found in T6. The analysis of the bacterial composition of the territories showed that T1, T3, and T5 exhibited the highest number of genera with 9, 5, and 4, respectively, with some genera common to several territories, such as *Bacillus* (T1, T2, T4, T5, and T6), *Stutzerimonas* (T1, T3, T4, T5, and T6), and *Acinetobacter* (T1, T3, T4, and T5). This analysis therefore shows the variability in the distribution of bacterial genera, with profiles that are both ubiquitous and specific depending on the region.

**Figure 4:**
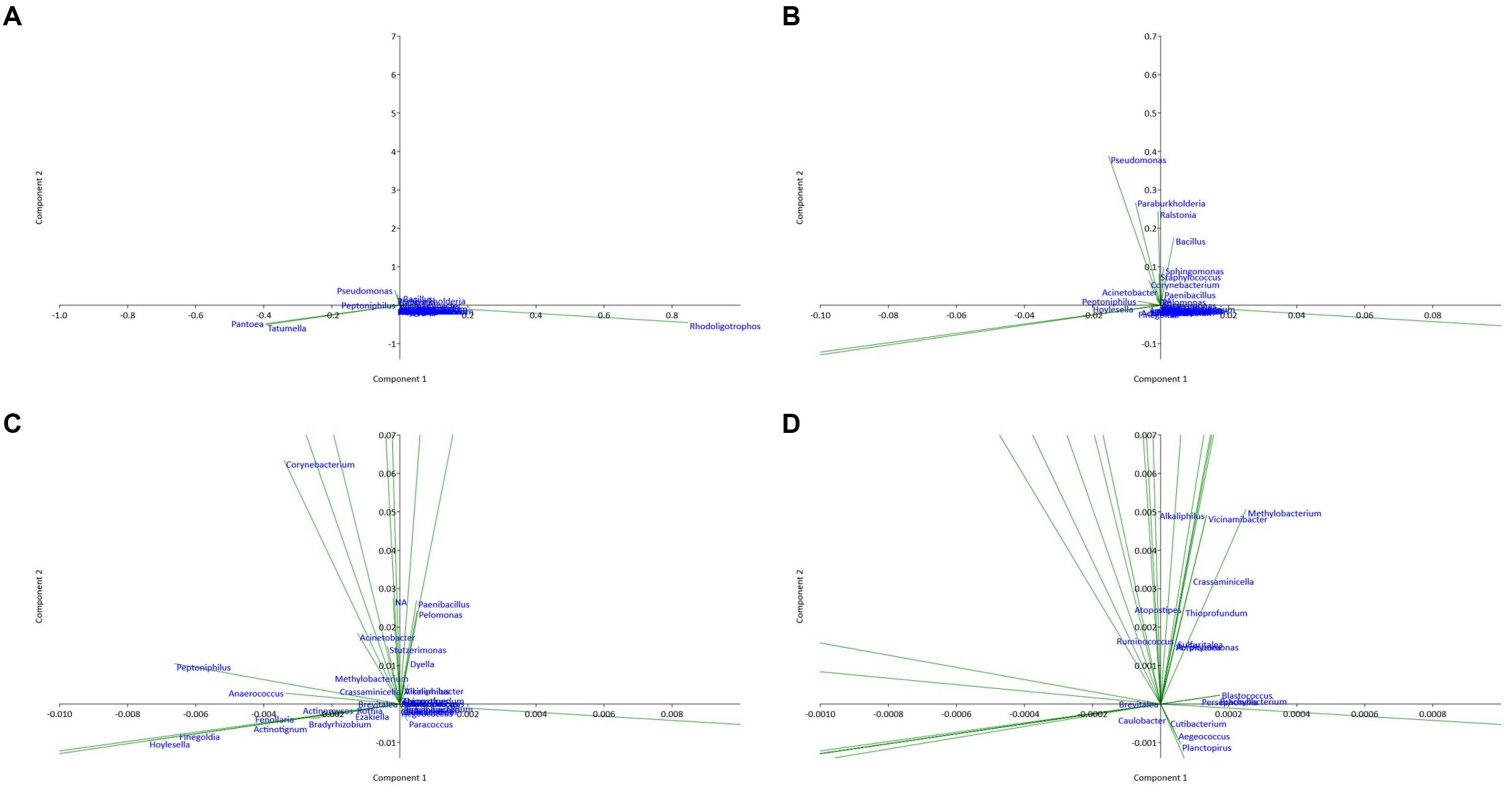
Principal component analysis (PCA) plot illustrating the correlation between bacterial communities of the six territories with different scales: **(A)** overview; **(B)** X10 zoom; **(C)** X100 zoom; **(D)** X1000 zoom.

The results of the principal component analysis (PCA) represented in Figure 4 show that the six studied sites differ significantly in terms of endophytic composition. The two principal axes PC1 and PC2 correspond to the largest proportions of variance in the data, and they facilitate the visualization of dissimilarities between the bacterial communities across sites. PC1 and PC2 summarize the major sources of variance, with sites featuring comparable relative abundances of bacterial taxa clustered more proximally on the biplot. The separation of sites along the principal component axes indicates that differences in the relative abundances of specific bacterial taxa drive the observed dissimilarities between communities. Concerning the bacterial community, sites T2, T4, T5, and T6 showed positive coordinates on the PC1 axis, suggesting that they have similarities in terms of endophytic composition. When the sites T1, T2, and T4 have positive coordinates on the PC2 axis, they also have similarities but differ from the other sites. In contrast, site T3 has negative coordinates on the first two principal axes, suggesting that it is different from the other sites in terms of endophytic composition. Several bacterial genera had high coordinates on the first two principal axes. For example, the presence and abundance of *Alkaliphilus* are positively correlated with the PC1 axis, while the genera *Pseudomonas and Tatumella* have high negative coordinates on this axis. Similarly, the bacterial genera *Bacillus, Paenibacillus, Pelomonas, Sphingomonas,* and *Staphylococcus* show high coordinates on the PC2 axis, which suggests that they are important in explaining the variability of the data on this axis. On the other hand, bacterial genera with relatively low coordinates on the first two main axes (PC1 and PC2) are less important in explaining the variability of the data. However, this does not necessarily mean that they do not play an important role in the endophytic composition of the sites studied. These results suggest that the bacterial composition of argane seeds varies considerably depending on the site, with significant differences in the presence and abundance of bacterial genera. This variation may have important implications for the health and growth of the argane tree, as well as for the development of biological control strategies against the diseases and pests that affect this species.

#### 3.1.2 Fungal community

**Figure 5:**
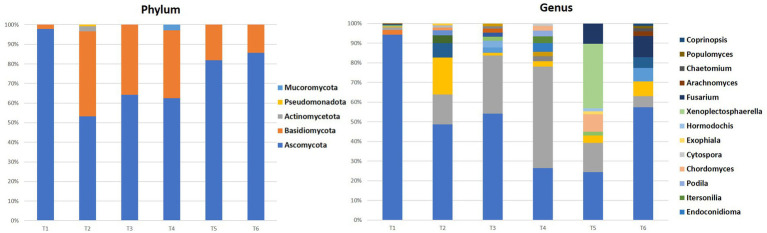
Percentages of phylum and the 10 most representative fungi genera in all samples.

Fungal communities play also important roles in seed health and can vary significantly between environmental niches. Figure 5 provides a visual summary of the relative abundances of major fungal phyla and genera in each seed sample.

The fungal communities of the majority of the sites (T1, T2, T3, T5, and T6) were dominated by the Ascomycota phylum with values between 53 and 98% of all fungi, followed by Basidiomycota (2–43%), except for the site T4 where the Basidiomycota phylum represents 63%, followed by Ascomycota with 35% of all fungi. The latter could be explained by the particular environmental conditions that favor these fungi.

In the genus level, *Penicillium* dominated most of the territories with a percentage varying between 46 and 94% of all fungi, except for the T4 and T5 where it displayed values ranging between 23 and 26%. Other genera such as *Alternaria, Malassezia, Cladosporium, Aspergillus, Clonostachys, Phanerochaete, Spathaspora, Rhodotorula, Neobulgaria, Dothiorella, Wallemia, Sporobolomyces, Aciditerrimonas, Aeromicrobium,* and *Devosia* were present with varying levels of abundance depending on the territory.

Similar to the bacteria, the differences in fungal community composition depending on the geographical origin of the seeds could have an impact on the properties of the seeds and the argane oil extracted from them. Further studies on the ecological role and interactions of these fungi with the argane tree would provide a better understanding of these associations. Overall, these results provide a good overview of the fungal diversity in this original and economically important system, the argane grove.

**Figure 6:**
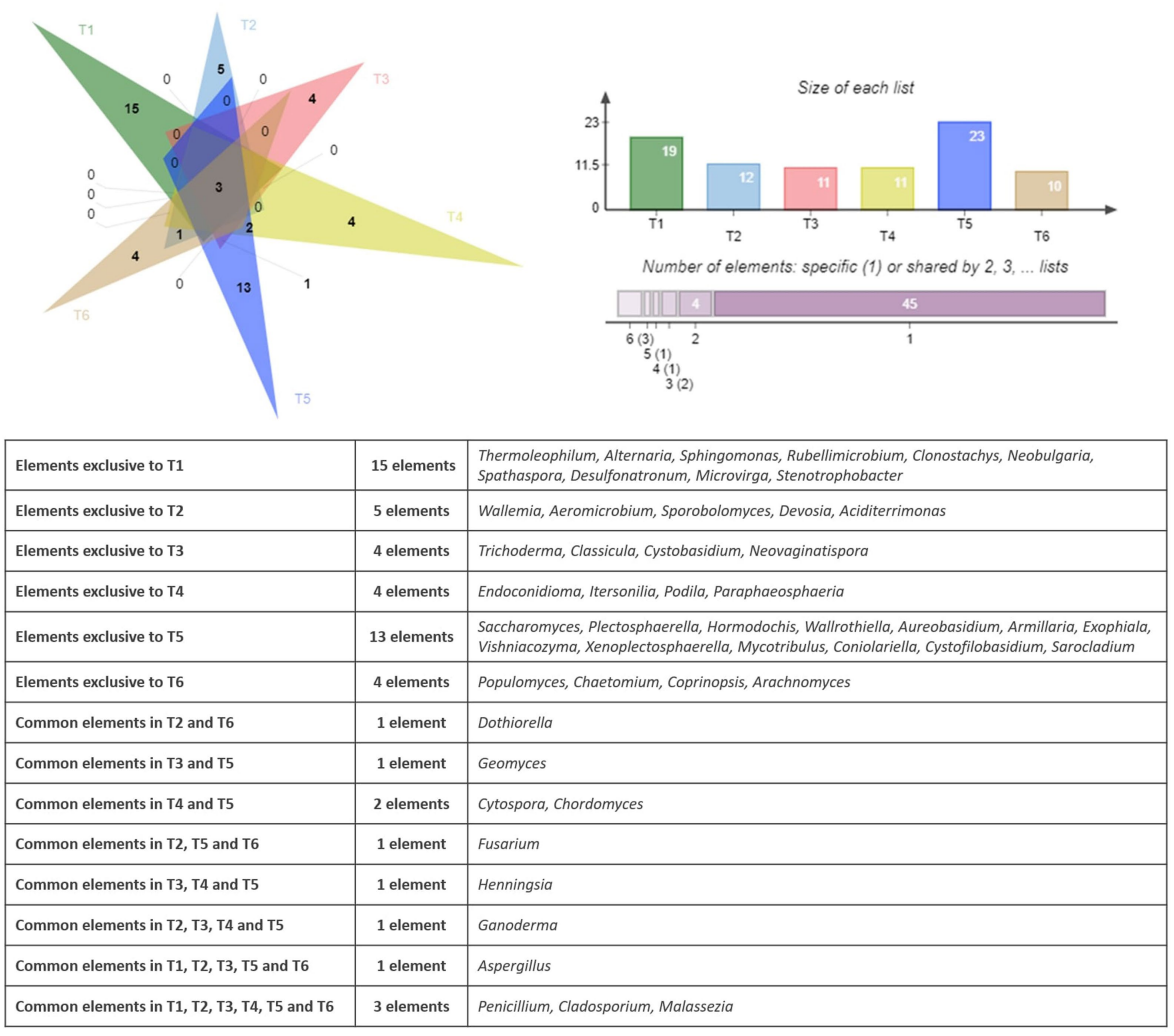
Venn diagram showing the specific and common genera in the fungal community.

The similarities and differences between the fungal communities had been displayed using a Venn diagram. Figure 6 shows the shared and unique fungal genera identified in the endophytic microbiome of argane seed samples collected from six different territories.

**Figure 7:**
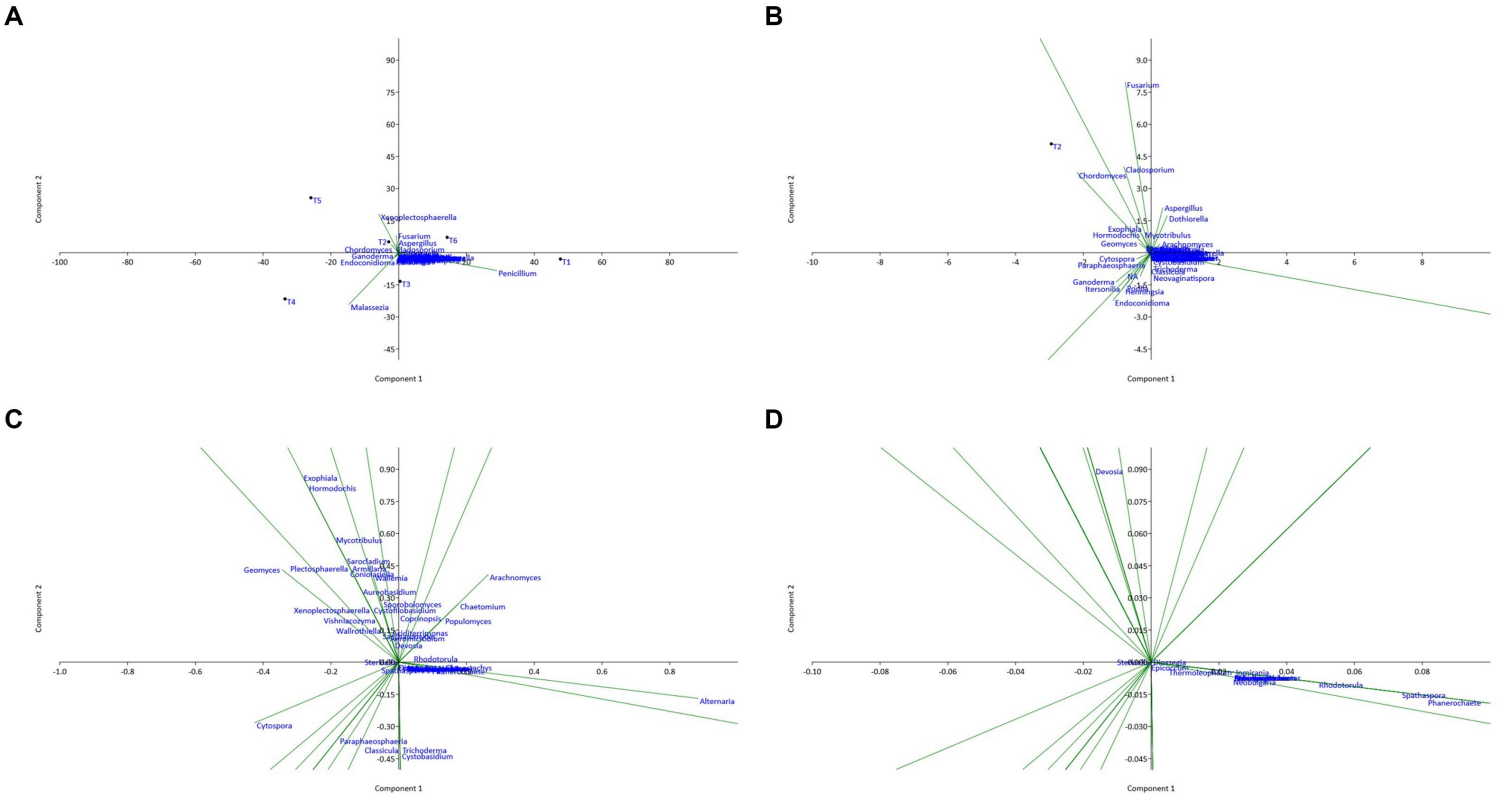
Principal component analysis (PCA) plot illustrating the correlation between fungal communities of the six territories with different scales: **(A)** overview; **(B)** X10 zoom; **(C)** X100 zoom; **(D)** X1000 zoom.

The analysis of the fungal genera distribution in the six sites (T1 to T6) reveals both ubiquitous genera and genera specific to certain territories. The sites T1 and T5 have the highest number of unique genera, with respective values of 15 and 13. It is also important to mention that some genera are exclusive to some territories, such as *Thermoleophilum, Alternaria, Sphingomonas, Rubellimicrobium, Clonostachys, Neobulgaria, Spathaspora, Desulfonatronum, Microvirga,* and *Stenotrophobacter* present on the site T1 and *Wallemia, Aeromicrobium, Sporobolomyces, Devosia,* and *Aciditerrimonas* reported on the site T2. Three fungal genera are present in all areas, namely *Penicillium, Cladosporium, and Malassezia.* Some genera are present in only 2 to 4 territories; for example, *Geomyces* is present in T3 and T5, *Cytospora* and *Chordomyces* are identified in T4 and T5, and *Dothiorella* is observed in T2 and T6. Other genera are specific to particular territories, such as *Aspergillus* found in T1, T2, T3, T5, and T6, as well as *Ganoderma* observed in T2, T3, T4, and T5. Similar to the bacterial community, a PCA had been applied to the results of the bacterial community. Figure 7 represents the PCA plot of the fungal community of the six territories (T1 to T6).

The analysis of the PCA plots shows that sites T1 and T6 have positive coordinates on the PC1 axis, while sites T4 and T5 have high negative coordinates on this axis. This suggests that sites T1 and T6 are similar in terms of endophytic composition, as well as sites T4 and T5. In contrast, sites T2 and T3 have relatively low coordinates on the PC1 axis, suggesting that they are different from the other sites in terms of endophytic composition.

Moreover, sites T2 and T5 have positive coordinates on the PC2 axis, while sites T3 and T4 have high negative coordinates on this axis. This suggests that sites T2 and T5 are similar in terms of endophytic composition, as are sites T3 and T4. Sites T1 and T6 have coordinates close to zero on the PC2 axis, suggesting that they are different from the other sites in terms of endophytic composition on this axis.

Several fungal genera have high coordinates on the first two principal axes. For example, the presence and abundance of the genera *Penicillium* and *Malassezia* are positively correlated with the PC1 axis, while the genus *Fusarium* has high negative coordinates on this axis. Similarly, the genera *Chaetomium, Cladosporium, Dothiorella, and Aspergillus* show high coordinates on the PC2 axis, suggesting that they are important in explaining the variability of the data on this axis. Genera with relatively low coordinates on the first two main axes (PC1 and PC2) are less important in explaining the variability of the data. However, this does not necessarily mean that they do not play an important role in the endophytic composition of the sites studied. Additionally, the analysis of the genus on the first two principal axes of the PCA enabled us to identify the genus most important in explaining the variability of the data. The genera *Penicillium and Fusarium* had significantly high coordinates on the PC1 axis, while the genera *Aspergillus* and *Cladosporium* had high coordinates on the PC2 axis.

Finally, the PCA results showed that the genera most important in explaining the variability of the data were different from one site to another. Certain genera, such as *Penicillium, Malassezia, Fusarium, Aspergillus, and Cladosporium*, were present on all the sites studied, while other endophytic species were specific to certain sites. These results suggest that the fungal composition of argane seeds varies considerably depending on the site, with significant differences in the identified bacterial species. This variation may have important implications for the health and growth of the argane tree, as well as for the development of biological control strategies against the diseases and pests that affect this species.

### 3.2 Analysis of the microbial diversity

#### 3.2.1 Bacterial community

**Figure 8:**
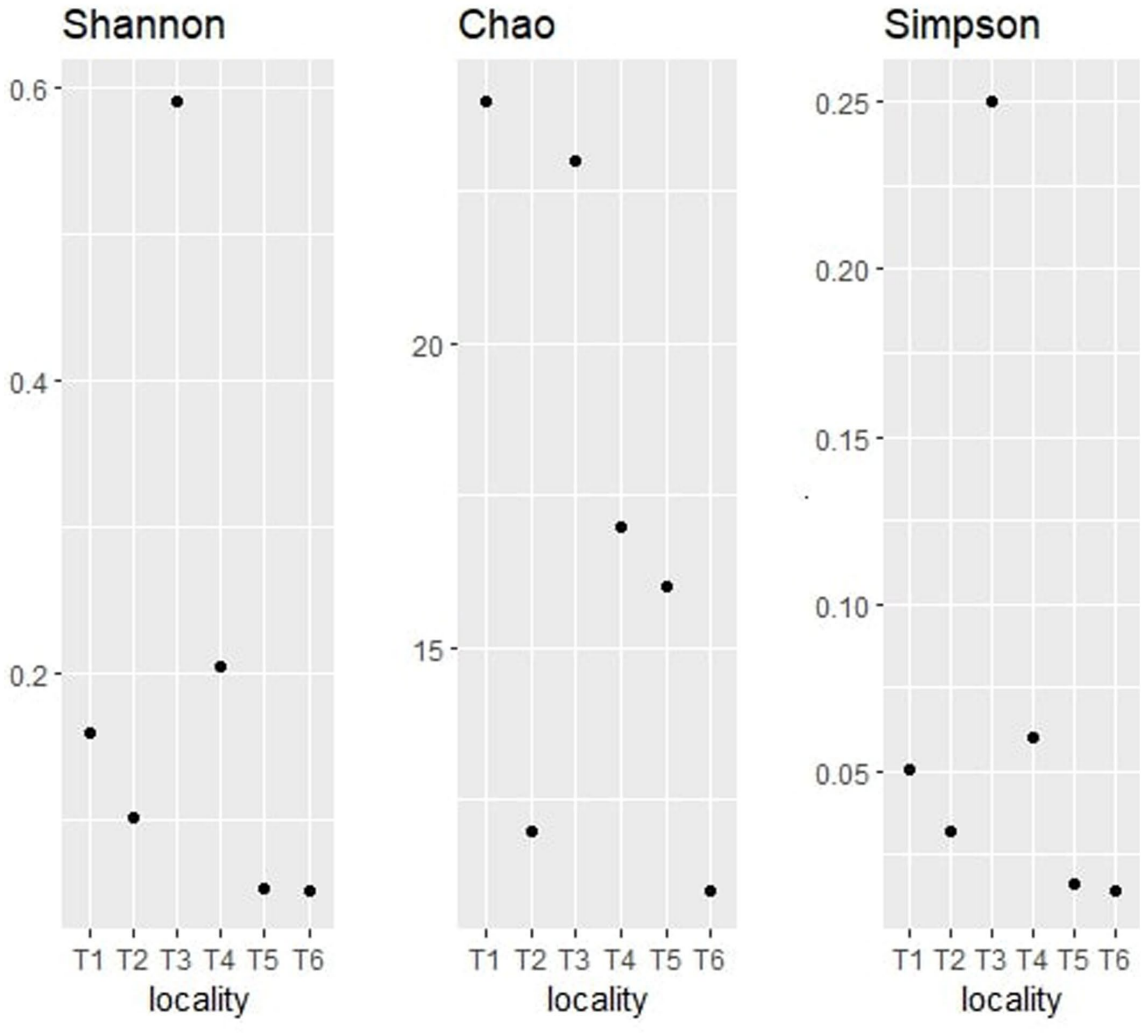
Alpha diversity indices (Shannon, Chao, and Simpson) for bacterial taxonomy.

The alpha diversity indices (Shannon, Chao, and Simpson) of the bacterial community are represented in Figure 8.

The Shannon index is one of the most widely used measures of alpha diversity (Thukral, 2017). It takes into account both the number of species present (richness) and the evenness of their distribution (relative abundance). The observation of the bacterial community shows that territory T3 has the highest Shannon index value with 0.5909, which indicates that it contains a high species richness and a high relative abundance of these species, followed by T4 with 0.2329 and T1 with 0.1586. On the other hand, territories T5 and T6 exhibited the lowest Shannon index values with 0.05297 and 0.05097, respectively. Those results suggest that T3 has a relatively high abundance of species, followed by T4, T1, T2, T5, and T6.

The Chao graph of the bacterial community indicates that territory T1 has the highest Chao1 index value with 24, indicating high species richness, followed by territories T3, T4, and T5 with 21, 17, and 16, respectively. The lowest Chao1 index values were T2 and T6 (lower than 10), suggesting low species richness.

The bacterial Simpson graph shows that territory T3 has the highest Simpson index value with 0.2505, followed by T4 and T1 with 0.07896 and 0.0528, respectively. T2, T5, and T6 showed the lowest values (inferior to 0.05). Those findings indicate that T3 is the most diverse in terms of species, with a more even distribution of species.

These differences may be explained by the variability of environmental factors, such as nutrient availability, temperature, pH, salinity, and humidity. For instance, territory T1 could have a higher availability of nutrients, which would favor the growth of a greater number of species. By combining the results of the three alpha diversity indices, we can conclude that territory T3 is the most diverse in terms of species. Territory T3 has the highest Shannon index value, indicating a high species richness and a high relative abundance of these species. This territory had also the lowest Simpson index value, suggesting a more equitable distribution of species in the sample. Finally, although territory T1 also has a high Shannon index value, it has a higher Simpson index value than territory T3, indicating a dominance of one or more species in the sample. Conversely, territories T5 and T6 have the lowest values for all alpha diversity indices, suggesting low species richness and dominance of one or more species in the sample. Territory T2 also has a low Chao1 index value, indicating low species richness. Overall, the alpha diversity analyses were useful for comparing bacterial endophyte communities between argan seed sources and inferring how location impacts community assembly.

#### 3.2.2 Fungal community

**Figure 9:**
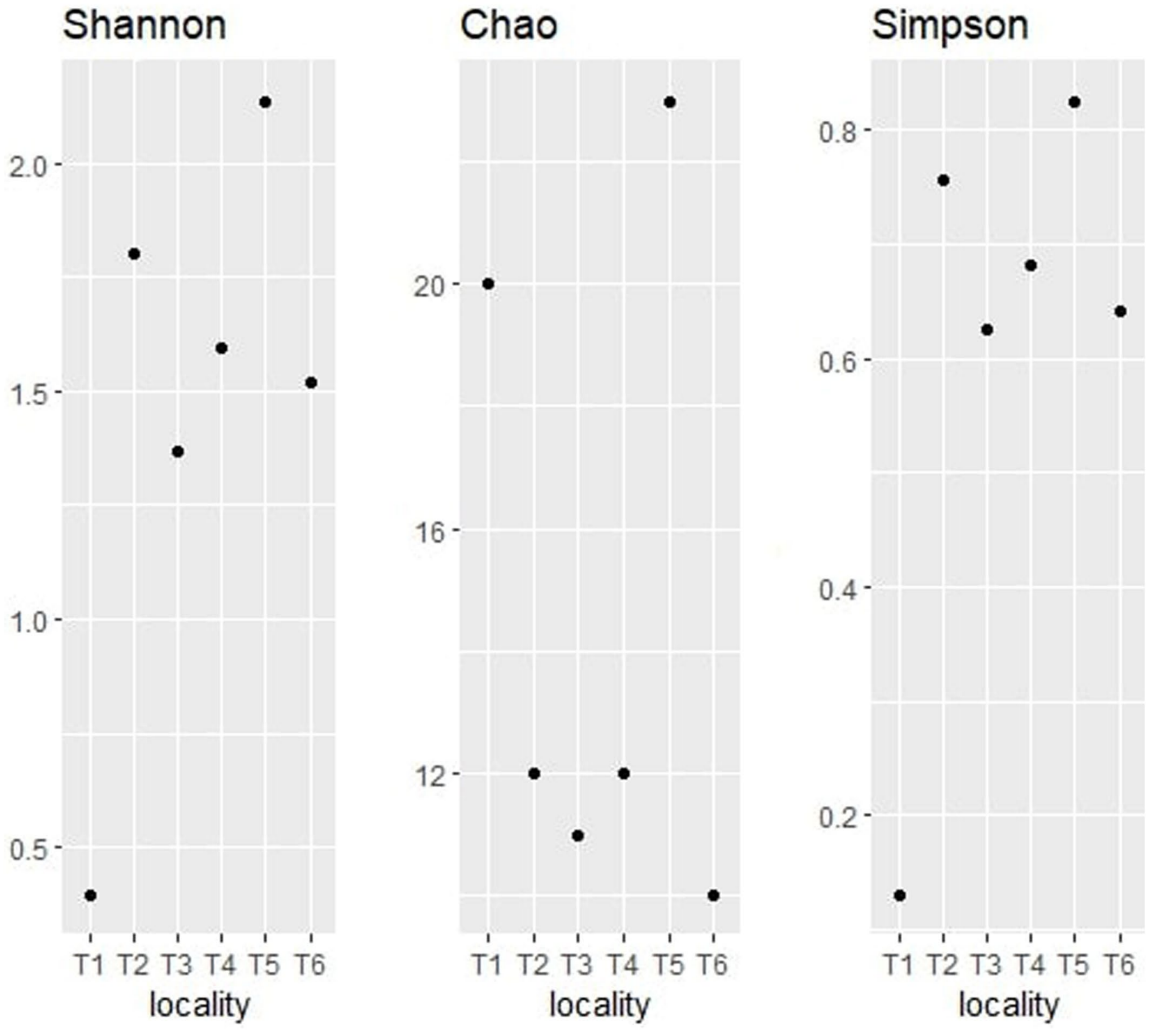
Alpha diversity indices (Shannon, Chao, and Simpson) for fungal taxonomy.

The alpha diversity indices, namely Shannon, Chao, and Simpson had also been determined for the fungal communities of the six territories. Figure 9 regroups the three calculated index representations of the fungal communities identified on the argane seeds.

The observation of the obtained alpha diversity indices of the fungal communities shows that territory T5 has the highest Shannon index value at 2.14, followed by T2 at 1.80. These values indicate a high richness in fungal endophytes and a high relative abundance of these genera. The lowest value was observed in T1 with a Shannon index value of 0.40.

Concerning the Chao1 index, T5 territory had the highest index value with 23, while T3 displayed a value of 20. Territories T2 and T4 showed a chao1 index value of 12, while T3 and T6 showed the lowest values (inferior to 12). The analysis of those results indicates that T5 and T1 are the highest in terms of richness of fungal endophytes, unlike T6 which seems to have the lowest fungal genera richness.

With regard to the Simpson index, it can be seen that territory T5 has the lowest Simpson index value with 0.83, directly followed by T2, T4, T6, and T3 with values ranging between 0.8 and 0.6, and then T1 with the lowest value (0.13). The analysis of those results shows that the samples T5, T2, T4, T6, and T3 are the most diverse in terms of fungal endophytes, with an important distribution of endophytes, while T1 is the least diverse in terms of fungal endophytes, with a strong dominance of one or more species.

The comparison of the three alpha diversity indices shows that T5 is the most diverse territory in terms of fungal endophytes. In fact, this territory has the highest Shannon index value, indicating a high richness of fungal endophytes and a high relative abundance of these endophytes. It also has the highest Simpson index value, suggesting a more equitable distribution of endophytes in the sample, and an important Chao1 index, indicating a high richness in terms of fungal endophytes. The differences observed between the territories may be due to various environmental factors, such as soil composition, nutrient availability, temperature, and humidity, which may affect the quality of the soil.

## 4 Discussion

The interactions between plants and endophytes are dynamic and can range from mutualism to parasitism to commensalism (Hardoim et al., 2008; Porras-Alfaro and Bayman, 2011). Endophytes reside in a large number of plant species as a microbiome and have proven to positively influence plant growth and responses to pathogens (Rosenblueth and Martínez-Romero, 2006; Porras-Alfaro and Bayman, 2011).

New biotechnological applications of endophytic species, such as bioremediation and phytoremediation, are experiencing considerable growth (Li et al., 2012). Endophytes play a key role in plant health through three mechanisms: biofertilization, phytostimulation, and biocontrol (Bloemberg and Lugtenberg, 2001).

Previous studies have shown that the endophytes *Actinomucor, Penicillium, Nectria, Curvularia, Drechslera,* and *Myrothecium* promote host tolerance to various stresses (Leitão and Enguita, 2016; d’Errico et al., 2021; Xiong et al., 2021). In another study, many species of *Colletotrichum* and *Penicillium* distinguished themselves by their beneficial effects on plant growth under unfavorable conditions (Waller et al., 2005; Redman et al., 2011; Hamilton and Bauerle, 2012). Furthermore, Miransari (2012) reported that plants required less effort to synthesize abscisic acid, thus protecting cell progression in case of stress, with the plant water balance being achieved through treatment with endophytic *Penicillium* spp.

From another point of view, microorganisms are also capable of producing cellulases, pectinases, xylanases, and amylases, which play an important role in the decomposition of organic matter and mineralization of nutrients. The production of cellulolytic, lipolytic, and proteolytic enzymes was observed in almost all isolates of *Bacillus* and *Paenibacillus* from sugar beets, which is not surprising as most were endophytes. Indeed, it is established that these enzymatic activities of endophytes allow plant hosts to be protected against pathogenic microorganisms, through lysis of the latter’s cell wall (Glick, 2012).

Several authors have reported that endophytes would have various bioactivities. Mastretta et al. (2009) reported the ability of *Nicotiana tabacum* plants for bioremediation through inoculation of their seeds with endophytes. They showed increased vegetative biomass production under cadmium (Cd) stress as a heavy metal, and the Cd concentration in plant tissues was higher than in non-inoculated plants. These results proved the useful effects of endophyte-inoculated seeds on heavy metal accumulation and assimilation. Furthermore, Choi et al. (2008) reported that phosphate solubilization in wheat and rice was mediated by gibberellic acid produced by *Pseudomonas* sp., and in another study, *Bacillus* spp. were found to play a role in improving maize nutrition by increasing the accumulation of several nutrients (Bomfim et al., 2020).

The quantitative endophytic composition is determined by the calculation of the taxa numbers after NGS determination (Petrović et al., 2023), while the qualitative endophytic composition is determined by the calculation of diversity indices (AlSharari et al., 2022). The alpha diversity index is an important metric in ecological studies, and it provides insights into both community structure and biodiversity patterns (Thukral, 2017). The alpha diversity index measures the number of different species and their relative abundances within an ecological community or sample, capturing the diversity present at a local scale (Wright, 2002). Common indices such as species richness, Shannon index, Chao, and Simpson allow for standardized comparisons of diversity across communities (Roswell et al., 2021). A higher alpha diversity indicates a more robust and complex ecosystem that can withstand environmental disturbances (Thukral, 2017; Yang et al., 2022). The Chao index estimates the species richness of a sample based on the number of rare species observed (Thukral, 2017). The Simpson index is another index for estimating alpha diversity (Thukral, 2017); this index takes into account species richness and relative abundance. However, it focuses more on the dominance of the most abundant species in the sample (Morris et al., 2014). Despite the fact that the identification of many microbes remains difficult (Reed and Tanprasert, 1995), the advent of next-generation sequencing (NGS) and metagenomic methods has provided a better understanding of microbial diversity by bypassing the drawbacks of the culture-based approach (David et al., 2014; Taylor et al., 2014). In fact, NGS has the advantage of identifying microorganisms not detected or overlooked in traditional culture methods (Price et al., 2009).

There are studies on the endophytic composition of argane roots (Abdallah et al., 2019; Mapelli et al., 2020); in those studies, the authors examined the microbial diversity associated with argane roots in arid and semi-arid environments. Abdallah et al. (2019) applied DNA sequencing techniques to identify microorganisms in the soil and roots of the argane tree, while Mapelli et al. (2020) used fungal culture and morphological identification techniques to investigate the diversity of the endophytic fungal community associated with argane tree roots. Both studies showed a high microbial diversity associated with the argane tree roots, which may have beneficial effects on the tree’s growth and health in harsh environments.

Furthermore, many bacterial endophytes that can be found on the seeds play beneficial roles. For instance, *Rhodoligotrophos* have a certain capacity for adaptation and survival in different environments, as well as a potentially important ecological role in the regulation of biogeochemical cycles in different ecosystems (Fukuda et al., 2012; Deng et al., 2014; Liu et al., 2019). Although *Rhodoligotrophos* dominated with high percentages, we also observed the presence of other genera such as *Pseudomonas, Paraburkholderia, Ralstonia, Bacillus, Corynebacterium, Streptococcus, Pelomonas, Staphylococcus, Acinetobacter, and Pantoea*, with significant variations depending on the studied areas. In addition, these genera may also play a role in the plant’s physiology but not as strongly as the Rhodoligotrophos genus.

According to the literature, many studies have revealed that these bacteria offer various environmental and ecological benefits (Gupta et al., 2015; Bhatt et al., 2022; Ting et al., 2023). The involvement of endophytic bacteria in improving agricultural yields is currently well established. Endophytic bacteria, which live and grow inside plant tissues, have been intensively studied for disease control and stress reduction in many plants and can therefore be a beneficial tool to improve crop growth and production in stressful and non-stressful environmental situations (Singh et al., 2021). For instance, several studies have shown that *Pseudomonas* are effective in controlling *Phytophthora* spp. infections through the production of antibiotics, lipopeptides, and volatile compounds in different plant hosts (Carruthers et al., 1995; Tran et al., 2007; Hunziker et al., 2015). Additionally, many strains of *Pseudomonas* are known to antagonize *Xanthomonas citri* subsp. *citri*, the pathogen responsible for citrus HLB (Unnamalai and Gnanamanickam, 1984; Michavila et al., 2017; Villamizar et al., 2020). Consequently, it could be a potential biopesticide against citrus HLB (Trivedi et al., 2011; Pistori et al., 2018; Blacutt et al., 2020). Moreover, *Pseudomonas* can also play a role in maintaining the health of citrus roots and produce a broad spectrum of bioactive compounds, including antibiotics, volatiles, and growth-promoting substances, making them well-suited as biocontrol and growth-promoting agents (Weller, 2007).

*Bacillus,* another identified genus, can inhibit the growth of a large spectra of bacterial species, some of which are pathogenic but most of which play important roles in agriculture and the environment (Santoyo et al., 2012). Several *Bacillus* species are used as biopesticides to control pathogens and improve agricultural yields (Chausali and Saxena, 2022). In fact, *Bacillus* has been successfully applied to many crops such as tomato, wheat, soybean, and sunflower (Aloo et al., 2019). Adeleke et al. (2021a) suggest that this species could be used to develop biocontrol agents. In particular, species such as *B. subtilis, B. megaterium, B. cereus, B. pumilus, and B. amyloliquefaciens* have been studied as potential bio-inoculants against citrus diseases and other plant pathogens (Fira et al., 2018; Albayrak, 2019; Chen et al., 2020; Adeleke et al., 2021b). Similarly, other bacterial genera identified in our study such as *Lactobacillus* and *Staphylococcus* were reported in the literature as potential biocontrol agents (Al-Dohail et al., 2011; Kharazian et al., 2017; Quattrini et al., 2018; Alijani et al., 2019; Cebrián et al., 2020). For example, *Staphylococcus xylosus* showed a biocontrol effect against toxigenic molds in meat (Cebrián et al., 2020).

To better understand the origin of the endophytes of the seeds, it would be interesting for a future study to examine the bacterial communities in the rhizosphere and surrounding soil. The variable presence of certain endophytes from one seed generation to the next suggests that the composition of the endophytic community evolves over the genustions. However, some studies have shown that members of seed-associated microbiomes can be conserved across plant genustions (Hardoim et al., 2008; Truyens et al., 2016) and even through human evolution and selection (Johnston-Monje and Raizada, 2011).

Concerning the fungal community, *Ascomycota* represent the largest fungal phylum with over 93,000 species (Senanayake et al., 2022). Their identification in all cultivars is therefore not surprising, and the results obtained in this study are consistent with the study published by Rim et al. (2021). In their study, they also identified *Ascomycota* as the dominant fungal phylum with 91.06%, along with *Basidiomycota* (5.95%) and *Mucoromycota* (2.97%), in four species of Pinus in Korea (Rim et al., 2021). According to the literature, the soil *Ascomycota* is known to increase the ability of soils to decompose plant biomass and maintain soil stability, carbon, and nitrogen cycling (particularly in arid regions) (Challacombe et al., 2019).

In terms of genus, this study identified 61 important fungal genera including *Alternaria, Malassezia, Cladosporium, Aspergillus, Clonostachys, Phanerochaete, Spathaspora, Rhodotorula, Neobulgaria, Dothiorella, Wallemia, Sporobolomyces, Aciditerrimonas, Aeromicrobium,* and *Devosia*. It is interesting to note that several species of some of these genera, in particular, *Aspergillus* (Atehnkeng et al., 2008)*, Penicillium* (Droby et al., 2002), and *Cladosporium* (Islam, 2022), have been identified as biocontrol agents. According to the study published by Boughalleb-M’Hamdi et al. (2018), *Aspergillus flavus* and other species studied proved to be good biocontrol agents against soil-borne fungi of melon and watermelon. In another report by Atallah et al. (2022), *Aspergillus* species namely *A. japonica, A. flavus, A. pseudoelegans, and A. niger* exhibited fungicidal activity against white mold disease of soybean caused by *Sclerotinia sclerotiorum*. Similarly, three species of *Cladosporium, namely, C. uredinicola, C. cladosporioides, and C. chlorocephalum*, were studied as potential biocontrol agents and proved to have insecticidal activity toward the whiteflies *Bemisia* spp (Abdel-Baky, 1998). Studies have also shown that certain genera such as *Aspergillus, Cladosporium, and Penicillium* can be used as effective post-harvest treatment against food-borne fungi of melons and watermelons (Abdel-Baky, 1998).

Finally, the constitution and functions of the seed microbiome are complex processes affected by multiple environmental and host genetic factors (Hardoim et al., 2008; Johnston-Monje and Raizada, 2011). Nevertheless, our results suggest that seed-dominant endophytes could provide numerous benefits covering the initial germination and establishment needs of seedlings (Truyens et al., 2015) and improve photosynthesis, nutrition, and stress alleviation (Arshad et al., 2007; Hardoim et al., 2008). The alpha diversity index is an important metric in ecological studies, and it provides insights into both community structure and biodiversity patterns (Thukral, 2017). The alpha diversity index measures the number of different species and their relative abundances within an ecological community or sample, capturing the diversity present at a local scale (Wright, 2002). Common indices, such as species richness, Shannon, Chao, and Simpson, allow for standardized comparisons of diversity across communities (Roswell et al., 2021). A higher alpha diversity indicates a more robust and complex ecosystem that can withstand environmental disturbances (Thukral, 2017; Yang et al., 2022). The Chao index estimates the species richness of a sample based on the number of rare species observed (Thukral, 2017). The Chao graph of the bacterial community indicates that territory T1 has the highest Chao1 index value with 24, indicating high species richness, followed by territories T3, T4, and T5 with 21, 17, and 16, respectively. The lowest Chao1 index values were T2 and T6 (lower than 10), suggesting low species richness.

## 5 Conclusion

In conclusion, this study provides the first characterization and comparison of bacterial and fungal endophyte communities associated with argan seeds from different geographical locations in the central-western region of Morocco using a metagenomics approach. The results show a dominance of *Pseudomonadota* at the bacterial level and *Ascomycota* at the fungal level, as observed in other plants. However, a significant variability in endophytic composition was observed across sites, at both bacterial and fungal levels, in line with the influence of environmental conditions highlighted in other studies. Multivariate statistical analysis confirms the existence of site-specific endophytic profiles, suggesting the influence of pedoclimatic conditions on community selection.

Certain ubiquitous taxa found at all sites could play a global ecological role for the argane tree, via mechanisms that remain to be elucidated. At the same time, the presence of endophytic taxa unique to certain sites opens up prospects for their use as bio-inoculants targeted according to geographical origin. To the best of our knowledge, this study is the first report on the identification of argane seeds endophytes, as well as the comparison of the difference in endophytic composition between six different territories in the center of Morocco. These findings are very important for identifying the prominent microorganisms in argan seeds. However, it remains unlikely to correlate the presence of these microbial species with agromorphological characteristics. This study paves the way for future studies to isolate and characterize endophytic strains of interest, assess their interactions with the argan tree, and develop their use as biocontrol or growth-promoting agents. The perspective of our study is to expand the sampling size and scope, including phenological and agromorphological studies, to determine the potential roles of seedborne microorganisms on argan tree development. Finally, this study provides the first data on the diversity of argane seed endophytes and highlights their geographical variability, opening up promising prospects for the sustainable management of the argane grove.

## Data availability statement

The raw data supporting the conclusions of this article will be made available by the authors, without undue reservation.

## Ethics statement

The manuscript presents research on animals that do not require ethical approval for their study.

## Author contributions

HS: Data curation, Formal analysis, Investigation, Methodology, Software, Writing – original draft. MF: Conceptualization, Data curation, Investigation, Methodology, Software, Validation, Writing – review & editing. AK: Formal Analysis, Methodology, Writing – review & editing. JR: _. NE: Conceptualization, Data curation, Project administration, Resources, Supervision, Validation, Visualization, Writing – review & editing. ND: Funding acquisition, Methodology, Project administration, Resources, Software, Supervision, Validation, Writing – review & editing.

## Footnotes

^1^https://bioinformatics.psb.ugent.be/webtools/Venn

^2^http://www.bioinformatics.com.cn/static/others/jvenn_en/example.html
